# Spray Dried Extract of *Phormidium valderianum* as a Promising Source of Natural Antioxidant

**DOI:** 10.1155/2014/897497

**Published:** 2014-08-10

**Authors:** Dipan Chatterjee, Paramita Bhattacharjee, Gour Gopal Satpati, Ruma Pal

**Affiliations:** ^1^Department of Food Technology and Biochemical Engineering, Jadavpur University, Kolkata 700 032, India; ^2^Department of Botany, Ballygunge Science College, University of Calcutta, Kolkata 700 019, India

## Abstract

Microencapsulation of antioxidant-rich fraction obtained by supercritical carbon dioxide extraction (at 50°C, 500 bar with extraction time of 90 min, and flow rate of CO_2_ at 2 L/min) of lyophilized biomass of* Phormidium valderianum* was carried out in a spray dryer using maltodextrin and gum arabic. Microencapsulation conditions that provided the best combination of phytochemical properties such as antioxidant activity, phenolic content, and reducing power with reasonable powder yield were an inlet temperature of 130°C and wall material composition as maltodextrin: gum arabic = 70 : 30. Toxicological study reported that the Anatoxin-a content of this encapsulated powder was below the limit of detection of HPLC. Storage study established that encapsulation of this antioxidant-rich algal extract resulted in eight times enhancement of half-life (*T*_1/2_) values. The release profile of microencapsulated antioxidant-rich fraction from the encapsulated powder was found to follow first order anomalous transport kinetics. Therefore, this microencapsulated algal extract with minimum toxicity is a source of natural antioxidant and could have promising use as novel dietary supplement.

## 1. Introduction

Microalga species are one of the important natural sources of antioxidants, owing to high amount of bioactive components such as polyunsaturated fatty acids, *β*-carotene [1–4], sulphated polysaccharides (anti-virals), and sterols (antimicrobials) [5, 6]. Among the various microalgal species,* Botryococcus* sp. [7],* Chlorella* sp. [8, 9],* Dunaliella* sp. [10],* Nostoc* sp. [11],* Phaeodactylum* sp. [12],* Spirulina* sp. [13, 14],* Haematococcus* sp. [15],* Chaetoceros* sp. [9],* Porphyridium* sp. [16], and* Galdieria* sp. [17] are reported to be sources of antioxidants.

One of the lesser studied sources of microalgal antioxidants with lower cellular toxicity is* Phormidium* species where pressurized fluid extraction using hexane, ethanol, and water have been applied to obtain antioxidant-rich fractions [18]. Although these extraction procedures are efficient, green, and environmentally safe for extraction of bioactive ingredients, the major problem is associated with usage of toxic organic solvents such as hexane which poses environment and health hazards [13, 19]. For these reasons, the extracts obtained by the above procedures are not suitable for food and pharmaceutical applications. Alternatively green technology of supercritical fluid (SCF) extraction using GRAS status CO_2_ as extracting solvent could be employed for obtaining antioxidant-rich extract from algae. In our previous investigation, we have successfully extracted antioxidant-rich fractions from lyophilized biomass of* Phormidium valderianum* with minimal Anatoxin-a content employing supercritical carbon dioxide (SC-CO_2_) extraction. However, it was observed that these antioxidant-rich fractions have low storage stability and exhibit high sensitivity to light, heat, and oxygen [20]. Therefore, it is opined that if the algal extracts are protected using microencapsulation technology, prior to its use as nutritional/therapeutic supplement, it would demonstrate better stability and shelf-life. Also, the algal extracts in its native liquid form have disadvantages in portability and commercial viability for use as natural antioxidant sources. Microencapsulation of the algal extracts would circumvent these aspects [21, 22].

Microencapsulations of antioxidant-rich fractions from various sources are generally carried out in a spray dryer using maltodextrin and gum arabic as matrices. Han et al. [23] used these matrices for encapsulating antioxidant-rich fraction from squalene, while Pitchaon et al. [24] and Rocha et al. [25] employed similar matrices for encapsulating polyphenol-based antioxidants. In our previous investigation, we have successfully used these matrices for encapsulating antioxidant-rich fraction from clove buds and administered the same as a source of natural antioxidant in soybean oil [26].

The primary objective of the current investigation is to encapsulate the antioxidant-rich algal extract (having minimum Anatoxin-a content) obtained by SC-CO_2_, in maltodextrin and gum arabic matrices using spray drying. Optimization of the microencapsulation process parameters was carried out to obtain the powder with appreciable yield and phytochemical properties. The encapsulated powder was then characterized in terms of encapsulation efficiency and phytochemical properties such as antioxidant activity, phenolic content, and reducing power. Stability studies of the antioxidant efficacies of encapsulated and nonencapsulated algal extracts were carried out for a storage period of 60 days. The release kinetics of antioxidants from the encapsulated powder and the related mechanism has been investigated by fitting the release data into different models. This work reports for the first time on microencapsulation of antioxidant-rich fractions from* Phormidium valderianum* in maltodextrin and gum arabic using spray dryer.

## 2. Materials and Methods

### 2.1. Reagents

Speciality chemicals such as DPPH, TPTZ, BHT, and Anatoxin-a (99% pure) were procured from M/s Sigma, Munich, Germany; methanol, *n*-hexane, chloroform, dichloromethane, isopropyl alcohol, and sodium carbonate from M/s E-Merck, Mumbai, India. All chemicals, solvents, media, and buffers used in the work were of AR grade.

### 2.2. Microalgae Samples and Their Culture Conditions

The algal biomass of* Phormidium valderianum *(Delp.) Gomont was collected from Department of Botany, Calcutta University, Kolkata, India, which was cultured in an open air aerobic tank (360 mm × 360 mm × 400 mm) using artificial sea nutrient (ASNIII) medium (NaCl (25 g/L), MgCl_2_·6H_2_O (2 g/L), KCl (0.5 g/L), NaNO_3_ (0.75 g/L), K_2_HPO_4_·3H_2_O (0.02 g/L), MgSO_4_·7H_2_O (3.5 g/L), CaCl_2_ (0.5 g/L), citric acid (0.003 g/L), ferric ammonium citrate (0.003 g/L), EDTA (0.0005 g/L), Na_2_CO_3_ (0.02 g/L), and trace metal (0.001 g/L)) at 25 ± 2°C, in 16:8 h light-dark cycle under cool fluorescent light (20–30 *μ* Em^−2^s^−1^) [20, 27]. Algal biomass for our study was collected during the log phase of growth (14–21 days, after inoculation).

### 2.3. Preparation and Extraction of Antioxidant-Rich Fraction

The extraction of antioxidant-rich fraction was carried out using lyophilized algal powder (*d*_*p*_ = 0.5 mm), prepared by subjecting the algal biomass in a bench top freeze dryer (M/s Eyela, Tokyo, Japan) and successively screening the lyophilized mass through a set of standard sieves in a sieve shaker [20]. SC-CO_2_ extraction of antioxidant-rich extract from lyophilized biomass of* Phormidium valderianum* was carried out in a SPE-ED SFE 2 model of M/s applied separations, USA. 10 g of lyophilized algal powder (*d*_*p*_ = 0.5 mm) was charged into a 50 mL extraction vessel (SS 316). To obtain the extract with best combination of antioxidant activity, phenolic content, and reducing power along with reduced Anatoxin-a content, the SC-CO_2_ extraction was carried out at 50°C, 500 bar with extraction time of 90 min (static time of 60 min and dynamic time of 30 min), and flow rate of CO_2_ at 2 L/min [20]. The algal extract was collected in an amber colored collection vial in an ice bath. The extract was subsequently weighed and stored in an amber colored screw capped vial in an inert atmosphere of nitrogen at 4°C in dark until further analyses.

### 2.4. Evaluation of Antioxidant Activities and Other Phytochemical Properties of the Algal Extracts

The antioxidant activity of the algal extract was determined by measuring the radical scavenging activity of DPPH [28] and was expressed as IC_50_ values. The total phenolic content determined by Folin-Ciocalteu reagent was expressed as *μ*g gallic acid equivalent/g dry algae [29] and its reducing power determined in accordance with the method of Oyaizu [30] was expressed as mg BHT equivalent/g dry algae, using standard curves prepared for gallic acid and BHT, respectively.

### 2.5. Toxicological Studies

Since Anatoxin-a is one of the most significant cyanobacterial toxins present in* Phormidium *sp. [31], we have assayed for the presence of this toxin in encapsulated and nonencapsulated antioxidant-rich fractions using HPLC-Photodiode array (PDA) analysis in a C18 column (250 × 4.6 mm i.d., 5 *μ*m) of a Perkin Elmer 200 Series HPLC System (M/s Perkin Elmer, California, USA), in accordance to the method reported by Gugger et al. [31] with certain modifications. Isocratic elution was carried out using water/acetonitrile (97.5/2.5, v/v) containing 0.1% formic acid (pH 3.5) at a flow rate of 0.8 mL/min. The photodiode array UV detection was carried out in the wavelength range of 200–300 nm in which Anatoxin-a recorded an absorbance maximum of 227 nm. HPLC studies were thus performed at 227 nm, the area under the curve of Anatoxin-a (*t*_*R*_ = 3.5 min⁡) was recorded, and the amount of toxin present in the sample was determined accordingly from the standard curve prepared [20].

### 2.6. Microencapsulation of Algal Extract

In the current investigation, the microencapsulation of antioxidant-rich algal extract was carried out using Mini Spray Dryer B-290 (M/s Buchi, Switzerland) model in a matrix comprised of maltodextrin and gum arabic. Optimization of the encapsulation parameters such as composition of wall material, maltodextrin: gum arabic (80 : 20, 70 : 30, 60 : 40), and inlet temperature (170°C, 130°C and 85°C) was conducted using a 3-level full factorial design. Batch size of algal extract and other process parameters were set through several experimental trials. 50 mg algal extract was added to 100 mL solutions containing different proportions of wall materials. The emulsions were created by mixing the solutions in an Ultra-Turax homogenizer (M/s Ika, Germany) for 30 s, which did not require any additional emulsifier. The spray gas pressure, sample feed rate, and atomization pressure were kept constant at 6 bar, 1.40 mL/min, and −65 mbar, respectively. The powders obtained were packed in aluminum foils and placed in Ziploc pouches (M/s Johnson, India), flushed with nitrogen, and stored at 23 ± 2°C until further analyses.

### 2.7. Characterization of Encapsulated Algal Extracts

For quantifying the total bioactive compounds (TB), the material of the coating structure of the microcapsule was completely destroyed in accordance to the procedure reported by Robert et al. [32], with little modifications. 1 g of the encapsulated powder was accurately weighed and added to 10 mL methanol. The dispersion was agitated using a vortex (1 min) and then subjected to sonication in a probe sonicator (M/s Ika, Germany) for 30 min at 1,000 rpm. The solution was then centrifuged at 11,200 g for 5 min and finally filtered. The supernatant was analyzed for bioactive compounds such as antioxidant activity (IC_50_ values of DPPH assay), total phenolic content (*μ*g gallic acid equivalent/g powder), reducing power (mg BHT equivalent/g powder), and Anatoxin-a content. The amount of sample retained on the surface of microcapsules was also determined in accordance with the method adopted by Robert et al. [32], with slight modifications. 200 mg of encapsulated powder was treated with 20 mL of acidified mixture of ethanol and methanol (1 : 1). These dispersions were agitated in a vortex at room temperature for 1 min and then filtered using 0.45 *μ*m Millipore filter and then assayed for antioxidant activity (by DPPH assay), total phenolic content, and reducing power. The % surface binding (SB) was determined by the following equation:

(1) SB(%)=(Surface  bioactive  compoundsTheoretical  bioactive  compounds)×100.  

The microencapsulation efficiency (ME) was calculated according to (2) with the results of total bioactive compounds and theoretical bioactive compounds [33]:

(2) $$\begin{array}{c} \text{ME}\left(\%\right)=\left(\frac{\text{Total}\;\text{bioactive}\;\text{compounds}}{\text{Theoretical}\;\text{bioactive}\;\text{compounds}}\right)\times 100. \end{array}$$

### 2.8. Scanning Electron Microscopy

The outer structure of the encapsulated powder containing algal extract was studied by scanning electron microscopy (SEM). The sample was coated with gold using a Vacuum Evaporator (M/s Emitech, Netherlands) and analyzed using ESEM Quantum Mark II (M/s FEI, Netherlands) operated at 20 kV with a working distance of 9.5 mm. The scanned images were collected digitally using XT Microscope software.

### 2.9. Storage Study of the Antioxidant Activity of the Algal Extracts

Storage stabilities of the antioxidant potencies of the nonencapsulated and encapsulated algal extracts were carried out by calculating the specific half-life values (*T*_1/2_) of the extracts. The samples were stored in the dark at ambient temperature (23 ± 2°C) and their antioxidant efficacies were evaluated at an interval of 10 days for a maximum period of 60 days. *T*_1/2_ values of the extracts were determined by calculating the ratio of the antioxidant activity of nonencapsulated and encapsulated algal extracts on day 0 (*TA*_0_) and on day (*t*) (*TA*_*t*_). The natural logarithm of the ratio *TA*_0_/*TA*_*t*_ was plotted against storage time. The slope of the line through the origin obtained by connecting the data points was equated with *k*, from which (*T*_1/2_ = (ln 2)/*k*) was calculated [34, 35].

### 2.10. Determination of Release Kinetics of Antioxidants from Encapsulated Algal Extract

The release curve of antioxidants from encapsulated algal extract powder was obtained according to the procedure of Song et al. [36] with little modifications. 2 g of encapsulated powder was added to a glass beaker containing 50 mL of phosphate buffered saline (pH 7.0) and subjected to continuous stirring using a magnetic stirrer at 100 rpm in dark at room temperature (23 ± 2°C). The samples were collected at different time intervals for 3 h. The antioxidant activity of the samples was determined by DPPH radical scavenging assay and the release curve was obtained from percentage of released antioxidants (related to the total antioxidant content) with time. To study the kinetics and mechanism of antioxidant release, the release profile of the same was fitted with several kinetic equations, namely, zero order (cumulative percentage antioxidant released v/s time), first order (log cumulative percent antioxidant retained v/s time), Higuchi model (cumulative percentage antioxidant released v/s $\sqrt{\text{time}}$), Peppas model (log cumulative percentage antioxidant released v/s log time), and Hixson-Crowell's cube root model ((percentage antioxidant retained)^1/3^ v/s time) equations. The kinetic model that best fitted the dissolution data was deciphered by comparing the regression coefficient (*r*) values obtained from these models. From the Peppas model, the release exponent “*n*” was estimated to characterize the different release mechanisms. According to Peppas's equation, *n* = 0.45 indicates “diffusion-controlled release,” *n* = 0.89 shows “swelling-controlled release” or case II transport, and “*n*” in the range of 0.45–0.89 indicates superposition of both the above phenomena and is known as “anomalous transport” [36].

### 2.11. Statistical Analysis

In this work, statistical analysis such as one-way ANOVA was conducted to study the effect of encapsulation parameters on the yield and phytochemical properties of the encapsulated powders. This statistical tool was also employed to know the significant differences in *T*_1/2_ values of antioxidant activities of encapsulated and nonencapsulated algal extracts. Significant differences between means were determined by Duncan's multiple-range test. A *P* value of 0.05 was used to verify the significance of all tests. All statistical tests of this experiment were conducted using STATISTICA 8.0 software (Statsoft, OK, USA) and MATLAB Version 7.6.0.324 (R2008a).

## 3. Result and Discussions

### 3.1. Phytochemical Properties of Nonencapsulated Algal Extract

At optimized SC-CO_2_ conditions (50°C, 500 bar, extraction time 90 min, and flow rate of CO_2_ at 2 L/min), the antioxidant activity of the algal extract (from the IC_50_ values of DPPH radical scavenging activity) was determined as 0.38 mg/mL. The phenolic content assayed was 97.21 *μ*g gallic acid equivalent/g dry algae and its reducing power was found to be 2554.71 *μ*g BHT equivalent/g dry algae. From the toxicological study, it was found that the Anatoxin-a content of algal extract obtained by SC-CO_2_ is 1.41 mg/g dry algae, which is 93% lower than the total Anatoxin-a content of the* Phormidium* biomass. All these results were found to be in close agreement with our previous investigation [20].

### 3.2. Characterization of Encapsulated Algal Powder

The yields of spray dried microencapsulated powder (containing antioxidant-rich algal extract) under different encapsulation conditions are presented in Table 1. The yield of the powder was found to increase with increasing inlet temperature and increasing concentration of maltodextrin and decreasing concentration of gum arabic in wall materials. The average yield of the powder was ~62%. Similar yields have been obtained in our previous study on encapsulation of clove extracts in maltodextrin-gum arabic matrix [26] and by other researchers who worked with the similar spray dryer model of M/s Buchi. While Maury et al. [37] reported 58% yield of spray-dried trehalose, Amaro et al. [38] obtained 60% yield of microparticles of sugar using the M/s Buchi model.

From the phytochemical properties of the encapsulated powder, it was found that the antioxidant activity, total phenolic content, and the reducing power of the powder decreased with increasing inlet temperature on account of thermosensitive properties of these bioactive compounds (Table 1). The powder containing low maltodextrin and high gum arabic retained better phytochemical properties than the one with lesser gum arabic content. The encapsulated powder produced at 85°C with maltodextrin: gum arabic = 70 : 30 as wall material, has maximum antioxidant activity (IC_50_ = 14.50 mg/mL) and phenolic content (180.82 *μ*g gallic acid equivalent/g powder), while the powder produced at the same temperature with maltodextrin: gum arabic = 60 : 40, has the best reducing power (9.13 mg BHT equivalent/g powder). However, the yield of the powder that exhibited best phytochemical properties was reasonably lower.

Encapsulation conditions that provided the best combination of phytochemical properties with considerable powder yield was at an inlet temperature of 130°C with wall material composition as maltodextrin: gum arabic = 70 : 30. These conditions were established as the optimum spray drying conditions for encapsulating antioxidant-rich algal extract obtained by SC-CO_2_. The encapsulated sample was then subjected to further characterization such as SEM analysis, storage study, and for assay of release kinetics of antioxidants.

**Figure 1:**
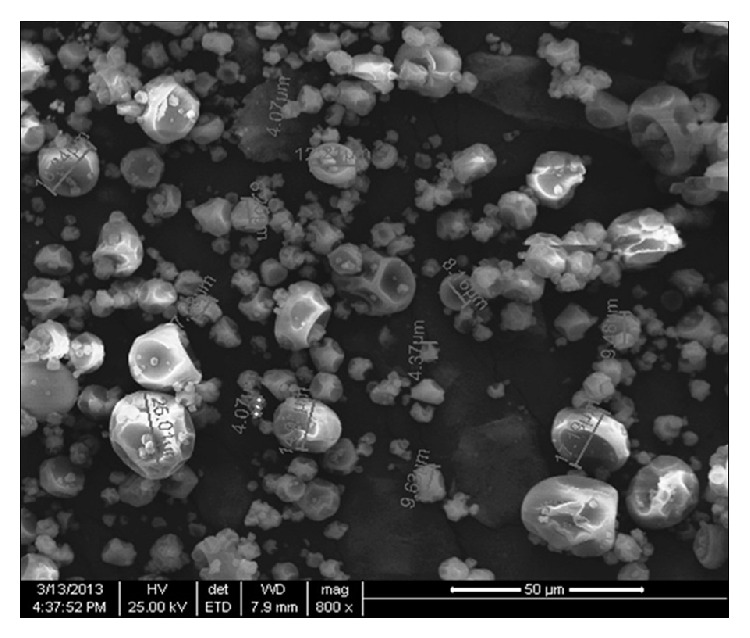
Scanning electron microscopy photograph of encapsulated powder containing antioxidant-rich algal extract obtained by SC-CO_2_ at a magnification of 800x.

From the SEM photograph of the encapsulated powder (Figure 1), it was found that the particles have shriveled morphology with size ranging from 2 to 30 *μ*m, with mean particle diameter of 11.57 *μ*m.

The microencapsulation efficiency of the bioactive compounds such as phenolic compounds and antioxidants were in the range of 60–65% and surface binding (%) of bioactive compounds varied between 25% and 30% (Table 1). Toxicological study reported that the Anatoxin-a content of encapsulated powder containing algal extract is below quantifiable limit, which indicates its amount in the encapsulated powder to be less than 1 mg/g dry algae. This is possibly due to the fact that very small amount of algal extract was utilized for encapsulation. Therefore, in the final product, that is, in the encapsulated powder, the Anatoxin-a quantity is below the quantifiable limit of HPLC. However, such low amount of algal extract contributes appreciable amount of antioxidant activity, phenolic content, and reducing power to the finished encapsulated product. These findings justify well the application of this encapsulated algal powder in food systems. Although seaweeds and several species of cyanobacteria are marketed as food supplements, they are known to contain significant amount of Anatoxin-a, which is not safe for human consumption [39, 40]. However, the encapsulated powder of antioxidant-rich fraction from* Phormidium valderianum*, obtained in this work could be used in food products and/or as a novel dietary supplement, owing to its high antioxidant activity, appreciable phytochemical potency, and minimum toxicity.

### 3.3. Storage Study of the Encapsulated and Nonencapsulated Algal Extracts

The changes in antioxidant activities of both nonencapsulated and encapsulated antioxidant-rich fractions of algal extracts over a storage period of 60 days are presented in Table 2. Storage stabilities of the same are depicted by their respective *T*_1/2_ values of antioxidant activities. It was found that *T*_1/2_ value of antioxidant activity of encapsulated algal extract at ambient temperature (23 ± 2°C) is 123.78 days which is significantly higher (*P* = 0.0000) than the *T*_1/2_ value of 14.82 days of nonencapsulated algal extract. Therefore, it could be summarized that encapsulation of antioxidant-rich algal extracts enhances the shelf-life of antioxidant activity eight times.

### 3.4. Release Kinetics of Antioxidant from the Encapsulated Powder

**Figure 2:**
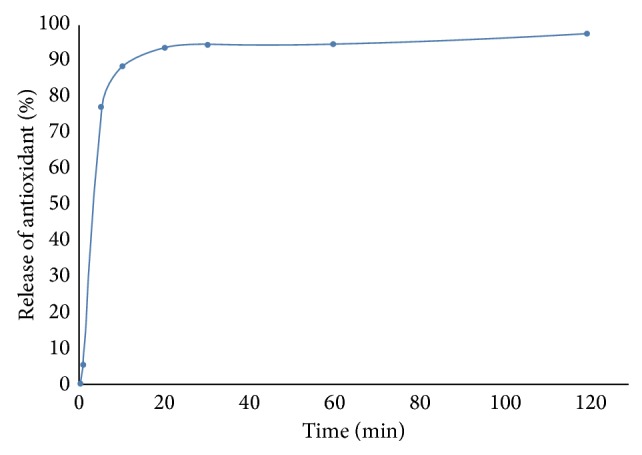
Release profile of antioxidant from encapsulated powder containing antioxidant-rich algal extract obtained by SC-CO_2_.

The release profile of antioxidants from encapsulated algal extract is shown in Figure 2. It was found that there was a strong burst effect in the first 20 min releasing ~90% of the antioxidant. This release profile was fitted to different kinetic equations and their corresponding regression coefficient (*r*) values were determined (Table 3). It was found that the “*r*” value of first order model was higher than their corresponding zero order models. Therefore, it could be concluded that the release profile of antioxidant-rich fraction obtained by SC-CO_2_ from encapsulated powder of maltodextrin and gum arabic matrix followed first order kinetics.

Among the different first order models studied, the “*r*” values for Peppas model was found to be higher than other models. Therefore, the release mechanism of antioxidant from the encapsulated powder was explained by the Peppas model using the following Korsmeyer and Peppas equation [41]:

(3) $$\begin{array}{c} \frac{M_{t}}{M_{a}}=K\times t^{n}, \end{array}$$

where *M*_*t*_/*M*_*a*_ is the fractional release of antioxidant in time “*t*,” “*K*” is a constant incorporating structural and geometric characteristics of the powder, and “*n*” is the diffusional release exponent indicative of the mechanism of release. The “*n*” value of encapsulated powder lies between 0.45 and 0.89 (Table 3); therefore, the antioxidant release behavior could be regarded as “anomalous transport” which is the superposition of diffusion-controlled release (Fickian diffusion) and swelling-controlled release phenomena (since maltodextrin and gum arabic are hydrocolloids) [36]. The value of “*K*” of encapsulated powder is 1.2. It may thus be established that the release of antioxidant from encapsulated powder comprising of maltodextrin-gum arabic matrix followed first order anomalous transport kinetics.

## 4. Conclusion

The current investigation has successfully encapsulated antioxidant-rich fraction obtained from SC-CO_2_ extraction of lyophilized biomass of* Phormidium valderianum* in a spray dryer. Microencapsulation conditions that provided the best combination of antioxidant activity, phenolic content, reducing power, and minimal Anatoxin-a content, along with considerable powder yield, were an inlet temperature of 130°C and wall material composition, maltodextrin: gum arabic = 70 : 30. The encapsulated powder had enhanced *T*_1/2_ values by eight times and the release profile of the antioxidants from the same followed first order anomalous transport kinetics. This powder could have promising use as a natural antioxidant.

## Units

 Extraction temperature (°C) (SI unit: K = C + 273.15)
 Extraction pressure (bar) (SI unit: Pa = 10^−5^ bar).

## Figures and Tables

**Table 1:** Yield and phytochemical properties of algal extracts encapsulated under different conditions.

Run number	Conc. of maltodextrin (%)	Conc. of gum arabic (%)	Inlet temperature (°C)	Outlet temperature (°C)	Yield of powder (%)^a^	IC_50_ of DPPH radical scavenging assay (mg/mL)^a^	Reducing power (mg BHT equivalent/g powder)^a^	Total phenolic content (*µ*g gallic acid equivalent/g powder)^a^	Surface binding of bioactive compounds (%)^a^
1	80	20	170	125	70.23 ± 1.10^a^	24.05 ± 0.27^a^	8.80 ± 0.78^a^	24.36 ± 1.12^g^	29.90 ± 0.28^a^
2	80	20	130	90	70.10 ± 0.98^a^	21.81 ± 0.24^a^	9.53 ± 0.69^a^	19.24 ± 0.98^g^	28.98 ± 0.10^a^
3	80	20	85	60	61.80 ± 0.76^ab^	18.85 ± 0.31^ab^	11.37 ± 0.42^a^	61.55 ± 2.13^e^	27.67 ± 0.33^a^
4	70	30	170	128	70.05 ± 1.12^a^	17.14 ± 0.56^ab^	7.08 ± 0.81^a^	107.72 ± 4.02^c^	27.18 ± 0.36^a^
5	70	30	130	95	62.8 ± 0.78^ab^	16.92 ± 0.71^ab^	7.30 ± 0.68^a^	146.19 ± 4.59^b^	26.73 ± 0.29^a^
6	70	30	85	62	57.55 ± 0.64^bc^	14.50 ± 0.97^b^	9.13 ± 0.26^a^	180.82 ± 6.83^a^	25.25 ± 0.47^a^
7	60	40	170	125	67.30 ± 0.67^a^	20.01 ± 0.33^ab^	8.54 ± 0.18^a^	7.69 ± 1.10^h^	28.34 ± 0.43^a^
8	60	40	130	95	54.60 ± 0.49^bc^	16.31 ± 0.81^ab^	11.77 ± 0.73^a^	41.04 ± 3.45^f^	26.28 ± 0.26^a^
9	60	40	85	64	49.93 ± 0.56^c^	15.75 ± 0.67^ab^	12.50 ± 0.59^a^	71.81 ± 4.12^d^	25.49 ± 0.45^a^

^a^Yield of encapsulated powder, IC_50_ values of DPPH radical scavenging assay, reducing power, total phenolic content, and surface bindings (%) of the encapsulated algal extracts are mean ± SD of three experimental runs.

Different letters in a column indicate significant difference at *P* < 0.05 level.

**Table 2:** Comparative analysis of antioxidant activity of encapsulated and nonencapsulated algal extract during storage period of 60 days.

Storage period (number of days)	IC_50_ of DPPH radical scavenging assay (mg/mL)^a^	
	Nonencapsulated algal extract	Encapsulated algal extract
0	2.36 ± 0.09^a^	16.53 ± 0.70^a^
10	4.99 ± 0.18^ab^	17.83 ± 0.68^ab^
20	5.66 ± 0.07^ab^	18.61 ± 0.81^abc^
30	9.83 ± 0.27^b^	19.65 ± 0.86^abc^
40	17.26 ± 0.56^c^	20.84 ± 0.92^abc^
50	24.91 ± 0.96^d^	22.04 ± 0.89^bc^
60	43.44 ± 0.89^e^	23.31 ± 0.97^c^

^a^IC_50_ values of DPPH radical scavenging assay are mean ± SD of three experimental runs.

Different letters in a column indicate significant difference at *P* < 0.05 level.

**Table 3:** Release kinetics of antioxidants from encapsulated algal extract.

Correlation coefficient (*r*)					Diffusional release exponent (*n*)	Release constant (*K*)	Type of transport
Zero order	First order	Higuchi	Hixon Crowell	Peppas			
0.54	0.76	0.77	0.67	0.85	0.51	1.20	Anomalous
